# Deciphering coccolith formation: advanced microscopy insights from the biomineralization of *Gephyrocapsa huxleyi*

**DOI:** 10.1098/rsif.2026.0369

**Published:** 2026-10-07

**Authors:** Alexander Triccas, Mariana Verezhak, Johannes Ihli, Manuel Guizar-Sicairos, Mirko Holler, Fraser Laidlaw, Martin R. Singleton, Virginie Chamard, Rachel Wood, Tilman A. Grünewald, Fabio Nudelman

**Affiliations:** ^1^ School of Chemistry , The University of Edinburgh, Edinburgh, Scotland, UK; ^2^ School of Physics and Astronomy, The University of Edinburgh, Edinburgh, UK; ^3^ Discovery Research Platform for Hidden Cell Biology, The University of Edinburgh, Edinburgh, UK; ^4^ School of GeoSciences, Grant Institute, The University of Edinburgh, Edinburgh, UK; ^5^ Max Planck Institute for Sustainable Materials, Max-Planck-Gesellschaft, Dusseldorf, Germany; ^6^ Photon Science Division, Paul Scherrer Institut PSI, Villigen, AG, Switzerland; ^7^ Swiss Light Source, Paul Scherrer Institut PSI, Villigen, AG, Switzerland; ^8^ Experiments Division, ALBA Synchrotron, Barcelona, Spain; ^9^ Institute of Physics, EPFL, Lausanne, VD, Switzerland; ^10^ Institut Fresnel, CNRS, Aix-Marseille University, Marseille, France; ^11^ Institut Fresnel Marseille, Marseille, Provence-Alpes-Côte d'Azur, France

**Keywords:** biomineralization, coccolith, calcite, crystallization, ptychographic tomography

## Abstract

Coccolithophores are unicellular marine phytoplankton that produce complex and intricately shaped mineralized scales called coccoliths. Coccoliths are produced in an intracellular vesicle where crystal nucleation occurs, from which several individual calcite units develop with anisotropic crystallographic facets, prompting studies into the cellular mechanisms that control crystal growth within the cell. Here, we characterize those morphological developments in three dimensions that occur during the formation of coccoliths by the species *Gephyrocapsa huxleyi* using cryo-ptychographic X-ray computed tomography. This technique is ideally suited to study coccolith mineral development, as intracellular structures can be imaged intact in their native state without needing to disrupt cells. Combined with additional imaging of developing coccoliths using cryo-transmission electron microscopy and scanning electron microscopy, we report a quasi-continuous sequence of coccolith growth with the respective calculated mass for each state. Within this growth sequence, we identified that a significant proportion of the coccoliths were at the proto-coccolith ring stage, consistent with a rate-limiting step in their formation that we correlated to the shift in growth of the crystals from isotropic to anisotropic, potentially owing to constraints imposed by adjacent crystal units. This study provides unique insights into the morphological evolution of coccoliths during their development, and strengthens our understanding of this important biomineralization process.

## 1. Introduction

Biomineralized tissues are essential to the survival of organisms across all six kingdoms, with their formation precisely controlled by manipulating the fundamental pathways of crystallization. By employing an array of cellular mechanisms, numerous aspects of crystal formation are modified: nucleation and growth stages, as well as morphology and polymorph selection [1–4]. As a result, complex-shaped crystals with hierarchical organization form, with vastly different structures than their synthetically grown counterparts. The ability of organisms to produce a range of functional mineralized structures with tunable properties from readily available ions attracts significant interest in materials science [5].

A model organism in the study of biomineral formation is unicellular marine phytoplankton called coccolithophores. These cells produce remarkable micrometre-sized scales with complex morphologies called coccoliths, displaying multi-faceted, intricately shaped crystals that form under strict biological regulation. Coccoliths consist of several interlocking single calcite subunits, synthesized in an intracellular vesicle where crystal nucleation and growth take place [6,7]. When fully mature, coccoliths are exocytosed from the cell, creating an external layer of interlocking scales called the coccosphere [8]. While coccolith formation is a well-studied process, many outstanding questions remain, particularly as to the conditions under which nucleation and growth are controlled in the intracellular vesicle. Crystallization pathways must also be resolved to determine the energetic costs involved in coccolith formation, which will largely affect how mineralization will respond to an increase in ocean temperature and acidity in light of anthropogenic climate changes [9–12]. This is of significant importance given the role that coccolith mineralization has in the global carbon cycle through its contribution to the formation of large geological carbon reservoirs [13].

Numerous studies have contributed to our understanding of how crystal growth and morphology are controlled in coccolithophores by defining the growth stages required to produce coccolith mineral. Initially, coccolith formation was studied by extracting undeveloped mineral units from cells and characterizing the crystal morphology at various stages in the vertical/radial (V/R) model [14–17]. Rhombohedral crystals first nucleate with their crystallographic *c*-axis orientated either vertically (V-units) or radially (R-units) aligned to an organic base plate [18,19]. Individual units then develop through mineral addition, with the expression of several specific crystallographic facets indicating that growth is precisely controlled. Several cellular mechanisms have been proposed to influence biological crystal formation in coccolithophores. Organic macromolecules are often cited as a major control factor of crystal growth. Polysaccharides extracted from mature coccoliths have been shown to promote anisotropic growth of specific crystallographic facets in synthetically produced calcite [20,21], while lattice distortions within the coccolith mineral indicate that macromolecules could template growth by blocking certain facets [22]. Growth is also known to be controlled through physical constraints created by the close confinement of coccolith subunits, with crystallographic facets dictated by the prevention of mineral expansion by nearby structures [23,24]. Additional control is imparted through the intracellular vesicle where mineralization occurs, both through physical confinement and alteration of ionic concentration gradients [25,26]. A feature of coccolithophore biomineralization is that each of the 200–250 classified living species produces coccoliths with completely unique organization and morphology of their crystal subunits [12]. It is likely that each coccolithophore species employs these growth control mechanisms to differing extents, reflected in their structural and morphological diversity.

Difficulties in defining these species-specific control mechanisms come from an incomplete understanding of how crystal morphology changes over the course of coccolith formation, particularly at early stages of growth. By focusing on the first stages of coccolith development in the species *Gephyrocapsa oceanica*, it was revealed that the vertical tube element is actually composed of two crystal layers, contrary to the one in the V/R model, tilted at opposite non-vertical angles relative to the base plate [23]. This adds complexity to the overall crystal morphology reported for this species and is an important feature which allows units to become tightly interlocked and influences the later formation of the shield elements. It is possible that these features develop owing to the highly constrained mineral growth space at these early stages, but this is difficult to clarify as undeveloped crystals are challenging to image in their native state.

Further characterization of coccolith morphology across the entire window of mineralization is therefore required to accurately determine the mechanisms involved in controlling crystal growth. To achieve this, entire cells of the coccolithophore *Gephyrocapsa huxleyi* (formerly known as *Emiliania huxleyi*) were imaged by cryo-ptychographic X-ray computed tomography (cryo-PXCT), which preserves cellular material in its native state and enables the three-dimensional reconstruction of coccoliths in their entirety [27,28]. As the field of view is sufficient to image around 80 *G. huxleyi* cells in an actively calcifying state, we were able to visualize a large number of coccoliths at multiple stages across their development. Through three-dimensional structural reconstructions, we report quasi-continuous growth across the entirety of mineralization. Additional characterization of crystal morphology evolution at early stages of coccolith development was provided by cryo-transmission electron microscopy (cryoTEM) and scanning electron microscopy (SEM). In sum, this allowed a precise depiction of the sequence of coccolith formation. We identified a rate-limiting stage during development that we correlate to the shift in crystal growth and morphology, from rhombohedral units growing isotopically to growing laterally over the neighbouring calcite crystal. Furthermore, we provide an estimation of the mineral mass of individual coccoliths, both fully formed and at different stages of their maturation. This research sheds light on the morphological evolution of coccoliths during development, deepening our understanding of this vital biomineralization process.

## 2. Experimental methods

### 2.1. Cultures

Cultures of *G. huxleyi* (920/9) were obtained from the Culture Collection of Algae and Protozoa (CCAP), Oban, Scotland. Cultures were incubated at 18°C on a 12 : 12 light : dark cycle. *Gephyrocapsa huxleyi* was grown in K/2 medium, adapted from Keller *et al.* [29], prepared using natural seawater from St Abbs Marine Station. Cultures were also supplemented with a Penicillin/Streptomycin/Neomycin Antibiotics solution (Fisher BioReagents). Cultures were maintained by subculturing 1 ml of culture into 30 ml fresh medium every two weeks. Cell growth was measured using a Leica optical microscope and a Neubauer improved haemocytometer.

### 2.2. Cryo-ptychographic X-ray computed tomography

#### 2.2.1. Preparation of *Gephyrocapsa huxleyi* cells

Cells were prepared by decalcifying 5 ml of culture in its exponential phase (5 days after subculturing) with 0.1 M pH 8.0 ethylenediaminetetraacetic acid (EDTA) to dissolve external coccoliths. Cells were left overnight in low-calcium (100 µM) Aquil artificial seawater, preventing further coccolith growth in order to recalibrate calcification. A 0.1 M CaCl_2_^.^2H_2_O (100 µl) solution was then added to raise [Ca^2+^] back to seawater levels (10 mM) and left for 24 h to provide sufficient time for coccolith production to resume while cell growth continued. Cells were harvested in 1 ml aliquots, centrifuged at 1500*g* and concentrated to an approximately 50 µl volume.

Capillaries with tips 10 µm wide were produced using a Flaming–Brown capillary puller (P-2000, Sutter Instruments) and mounted in hollow OMNY pins with UV resin [30]. The samples were loaded into the capillary tips from the accessible back side. The samples were then further sedimented into the very tip of the capillary by a small settling period or by mild centrifugation (2000*g* for 240 s). The capillaries were rapidly plunge-frozen in liquid ethane using a vitrification robot (FEI Vitrobot Mark VI). The sample chamber was maintained at 21°C and 100% humidity. Particular care was taken to minimize the time lag between sample preparation and sample freezing. The samples were stored at cryogenic temperature under liquid nitrogen and transferred to the beamline using a transport dewar to ensure a continuous cryogenic cold-chain, which was kept up until after the samples were measured in the OMNY instrument [31].

#### 2.2.2. Tomogram acquisition and reconstruction

Cryo-PCXT measurements were carried out at the cSAXS beamline (X12SA), Swiss Light Source, Paul Scherrer Institute, Switzerland with the OMNY instrument [31]. Ptychographic scans were performed at a photon energy of 6.2 keV under cryogenic conditions at 90 K. Doing the measurements under cryogenic conditions was important both for the preservation of the cellular material in its close to native state and to minimize radiation damage that is inherent to the large dose needed to produce a complete dataset. An Eiger 1.5 M detector, placed 7.2 m downstream of the sample, was used to collect far-field intensity patterns at each scanning point. The same ptychography projection scan was performed at various equiangular orientations, rotating the sample about the vertical axis in the range between 0° and 180°. The exact imaging parameters are listed in supplementary material, table S1.

The ptychographic reconstructions were carried out using 300 iterations of the difference-map algorithm, followed by 300 iterations of maximum-likelihood refinement [32], resulting in a reconstructed pixel size of 32 nm (supplementary material, figure S1) [33]. The projections were further processed and aligned using in-house developed MATLAB scripts (which are publicly available) [34]. Fourier shell correlation (FSC) was used to provide a first estimate of the spatial resolution. The datasets were split angularly into two separately reconstructed datasets and compared using the FSC approach [35]. In this way, the half-bit resolution was estimated as approximately 56 nm. The corresponding FSC curve is presented in supplementary material, figure S1.

### 2.3. Cryo-transmission electron microscopy

To extract intracellular coccoliths from *G. huxleyi* cells, 50 ml cell cultures early in the exponential phase (2 days after subculturing) were decalcified using 0.1 M pH 8.0 EDTA. The seawater medium was aspirated, and the pellet of decalcified cells was placed in a 0.1 M Na_2_CO_3_ solution (40 ml) overnight to cause lysis by osmotic pressure. The proto-coccolith ring, in particular at the early stages of formation, is not very robust and the crystals can easily fall apart. Preparation for conventional TEM would have required more extensive centrifugation and washing to remove excess of salts, and therefore could have caused damage to the structure. To minimize disruptions to the structure, cryoTEM was opted instead. The solution was concentrated down to a 20 µl volume and an aliquot (3 µl) of this coccolith-containing solution was applied to a cryoTEM grid and plunge frozen in liquid ethane at liquid nitrogen temperatures using a vitrification robot (FEI Vitrobot Mark VI). The sample chamber was maintained at 21°C and 100% humidity. CryoTEM grids (Au/C, R2/2 Quantifoil Micro Tools GmbH) were plasma treated for 1 min before freezing using a Quorumtech Glow Discharge system. An FEI Tecnai F20 electron microscope with a 200 keV field emission gun, equipped with a Gatan cryoholder operator at approximately –170°C was used for imaging. Images were recorded on a Gatan K2 Summit camera.

### 2.4. Scanning electron microscopy

For SEM imaging of mature *G. huxleyi* coccoliths and cells, 200 µl of culture in its exponential phase (5 days) was deposited on Isopore 0.2 µm polycarbonate (PC) membranes (Merck Millipore Ltd.). Cells were washed with 1 ml 8.0 pH dH_2_O to remove salt from the membrane and air-dried overnight. The membrane was mounted on SEM stubs and coated with Pt. For SEM imaging of intracellular coccoliths, cells were subjected to osmotic pressure using a 0.1 M Na_2_CO_3_ solution as described above for cryoTEM. A 200 µl aliquot of a concentrated coccolith solution was then deposited onto a 0.22 µm PC membrane. All cells and coccoliths were washed with 3 ml dH_2_O adjusted to pH 8.0 to remove salt from the membrane without dissolution of the mineral structures. All samples were coated with a thin layer of carbon before imaging. SEM imaging was performed on a Zeiss Crossbeam 550 cryoFIB-SEM with an accelerating voltage of 3 kV.

## 3. Results

### 3.1. Mapping morphological changes across coccolith development using ptychographic computed X-ray nanotomography

 To ensure cells were actively calcifying when cryogenically preserved, mineralization was induced by dissolving external coccoliths and then placing cells back in normal culturing conditions for 24 h. The resulting cells were directly frozen in solution and imaged by cryo-PXCT, which enables mineral structures and other dense features to be visualized and reconstructed in three dimensions (supplementary material, figure S2a–c and movie S1). From two-dimensional slices through a representative cell (figure 1a–f), a mineralizing calcite coccolith (red arrows in figure 1a–d) can be observed within, while several coccoliths (yellow arrows in figure 1a–f) surround the outer surface. Three-dimensional volume renderings of these features (figure 1g,h) reveal the intracellular coccolith (red in figure 1g,h) is not fully developed, in comparison with the extracellular coccoliths (yellow in figure 1h) that cover the cell surface. The morphology of the mineralized cell (figure 1i) appears to be consistent with those imaged by SEM (figure 1j), suggesting structural reconstructions from cryo-PXCT are accurate and that minimal damage occurred from cryogenic preparation. Electron-dense bodies can also be observed within cells (black arrows in figure 1f and black in figure 1g,h), similar in size and morphology to ion-rich bodies defined in this species [36]. Cryo-PXCT was used to report the variable morphology of similar bodies related to Ca storage and transport in the coccolithophore *Chrysotila carterae* [37]; however, we could not observe dense bodies with any consistent structural features or spatial relationship to the intracellular mineral structures in this study.

**Figure 1.**
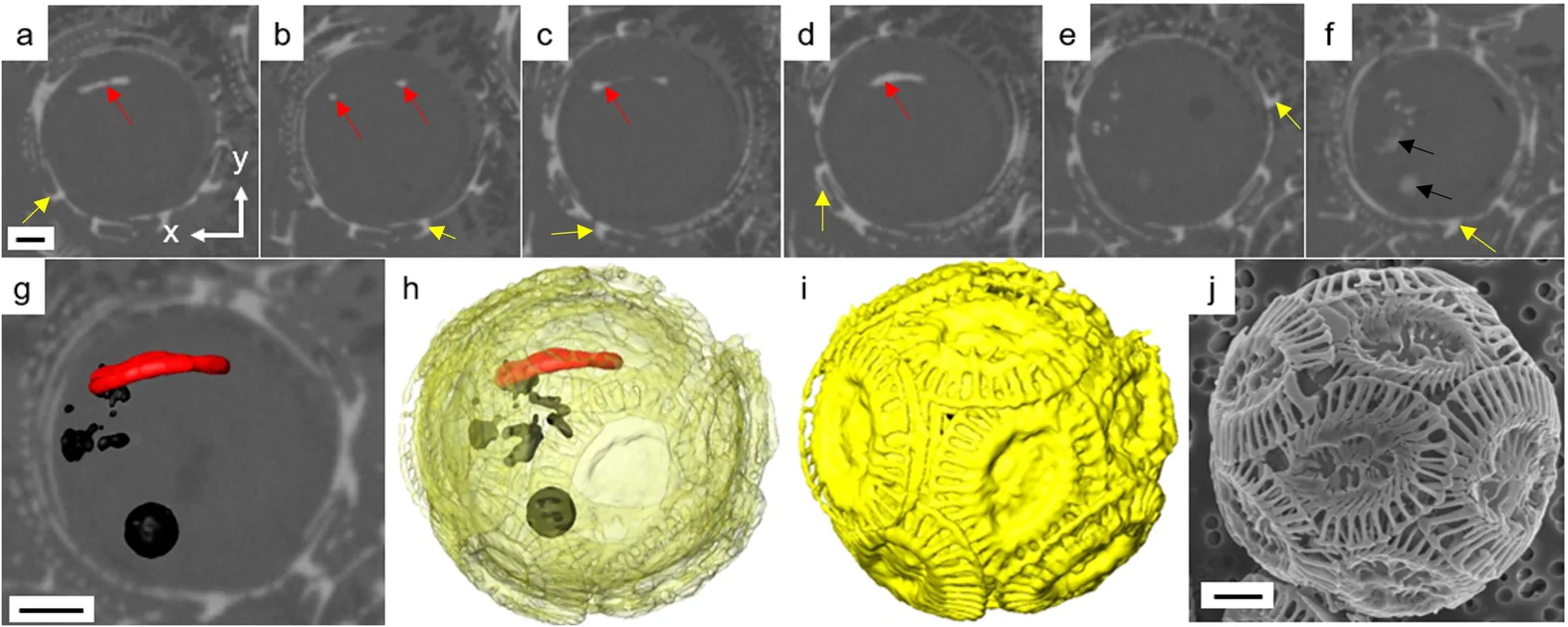
Cryo-PXCT of an actively calcifying *G. huxleyi* cell. Shown in (a–f) are orthoslices through one calcifying *G. huxleyi* cell, highlighting a forming intracellular coccolith (red arrows) and electron-dense bodies (black arrows), alongside several interlocked extracellular coccoliths (yellow arrows) on the outer cell surface. Provided in (g) are volume renderings of the intracellular coccolith (red) and electron-dense bodies (black) displayed in the orthoslices in (a–f). (h) Outside the cell, external coccoliths (yellow) cover the surface. The full coccosphere reconstruction is shown in (i), in comparison with a scanning electron micrograph of an intact cell (j). Scale bars: 0.5 µm.

From the population of *G. huxleyi* cells inside the capillary (supplementary material, figure S2a–c), a total of 88 intracellular coccoliths were identified. These coccoliths had distinct morphologies related to the stage of growth they were in when cryogenically preserved. Presented in figure 2 is a series of cross-sections and volume renderings of coccoliths selected to highlight the morphological transformations occurring across mineral formation, which are categorized here into five development stages based on clearly identifiable structural features and the assumption that a coccolith can only gain weight and volume during its growth. The smallest mineral structures which constitute stage 1 were flat spherical rings, possibly related to the first nucleation of the protococcolith ring. The smallest rings had a diameter as low as approximately 180 nm (stage 1a), but most were thicker, with a diameter between 250 and 300 nm (stage 1b). Mineral growth appears to be isotropic at this early stage. Of the 88 coccoliths analysed, 45 were at growth stage 1, with others distributed evenly across the later stages (supplementary material, table S1). Of the 45 coccoliths at growth stage 1, 6 were in stage 1a and 39 were in stage 1b. This suggests that the crystal growth occurring during this stage may be a rate-limiting step in coccolith formation.

**Figure 2.**
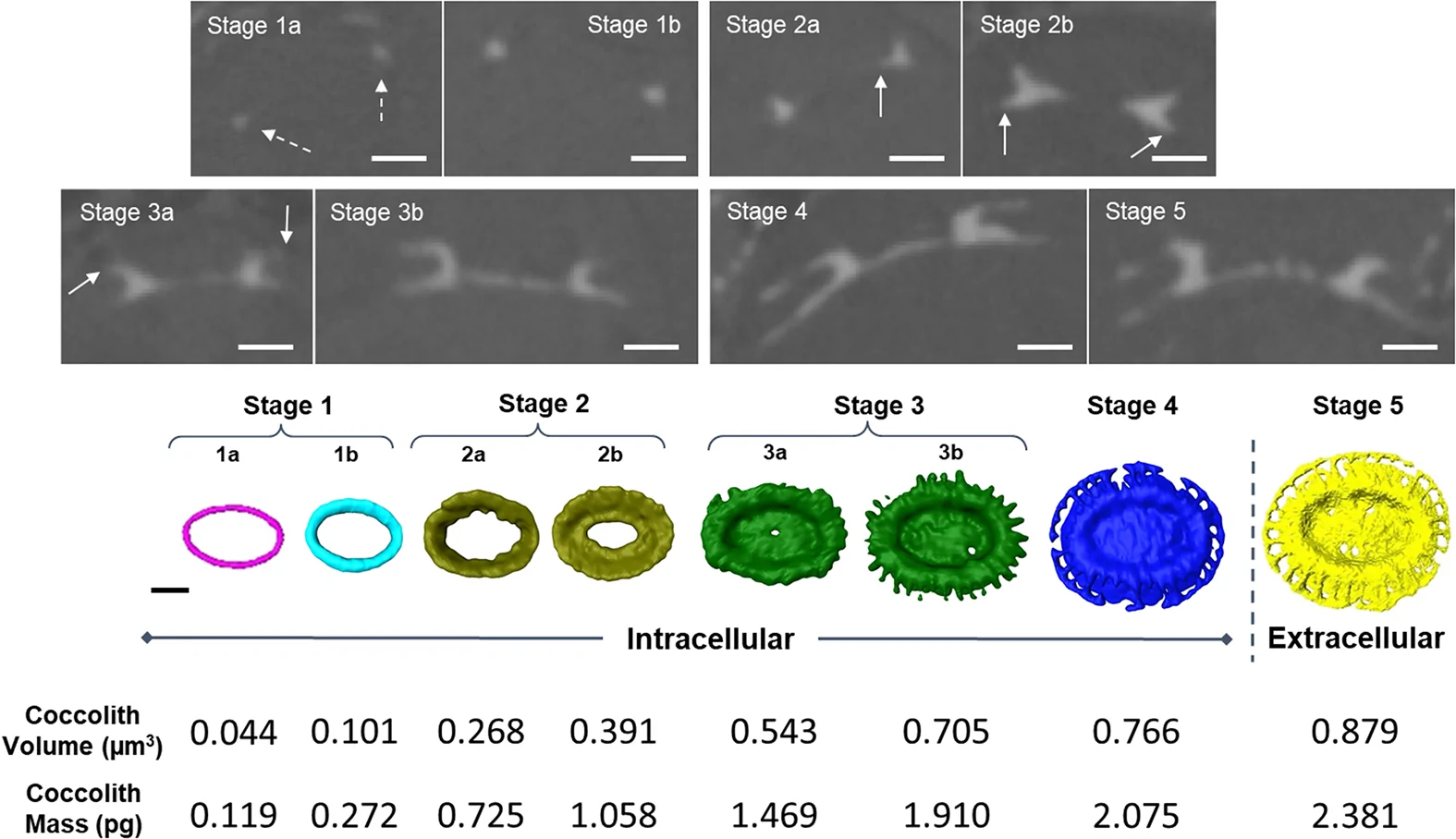
Morphological development of *G. huxleyi* coccoliths. Top: cryo-PXCT provides slices through the three-dimensional volume of coccoliths at various stages of development, showing morphological changes as the mineral becomes fully formed. Arrows indicate first appearance of: thin mineral ring in stage 1, central area elements in stage 2a, proximal shield in stage 2b, distal shield in stage 3a. Bottom: volume rendering of the eight coccoliths, with the calculated volume and mass of coccoliths at each stage of development. Scale bars: 0.5 µm.

Stage 2 is defined by preferential mineral addition to the top side of the spherical ring, relating to the vertical crystal growth that causes the tube element to form. Initial vertical growth results in a coccolith at stage 2a with a height of 300 nm. Additionally, small extensions also appear from the base of the inward-facing side of the mineral (arrow in stage 2a, figure 2), indicating the first appearance of the central area elements. Further vertical growth in stage 2b leads to the completion of the tube element, with a height of 400 nm. This stage also coincides with the coccolith tube expanding at its base, with outward addition starting the formation of the proximal shield (arrows in stage 2b, figure 2). In combination with this, larger mineral protrusions also appear on the inwards-facing side of the coccolith ring. These additions visibly change the coccolith morphology, which is now 500 nm wide at its base.

Stage 3 is defined by the presence of mineral regions which connect across the base of the coccolith tube, representing the completion of central area elements. Further identification of coccoliths at this growth stage was distinguished by new mineral regions at the top of the tube element (arrows in stage 3a, figure 2). Rather than continuing to extend vertically, the mineral curves around as growth changes to an outward direction, reflecting the formation of the distal shield elements. Both the proximal and distal shield elements then grow outwards in the same radial direction (stage 3b), both extending by approximately 200 nm from the coccolith tube.

Stage 4 is classified as fully developed coccoliths, with T-shaped distal shield elements. In a mature coccolith, the distal and proximal shield elements are, respectively, 680 and 800 nm in length. The thickness varied across both units, ranging between 30 and 120 nm. We also observed cells with two coccoliths being produced at the same time, in accordance with previous reports [38]. In all cases, one coccolith was always fully mature, while the other was either early in development around stage 1–2 (supplementary material, figure S3a) or nearing full maturation (supplementary material, figure S3b). Stage 5 is morphologically identical to stage 4, with coccoliths located outside the cell rather than inside, reflecting exocytosis of the scale rather than mineral growth.

PXCT also offers the advantage of providing electron-density values which relate to the composition of the structure imaged. Electron density can be converted into volume and mass, and as cells are imaged intact, the size and weight of coccoliths throughout their growth can be estimated. To calculate these values in light of the effect that resolution limitation has on identifying voxels belonging to the outer coccolith mineral regions, electron density values of all voxels comprising the coccoliths were summed together and divided by the expected electron density value for calcite mineral (0.82 n_e_ Å^-3^) [27]. This value was then multiplied by the size of a single voxel (3.28 × 10^−17^ cm^3^) to obtain the volume of the whole structure. From the volume of the coccolith, the mass can be calculated by multiplying by the calcite mass density (2.71 g cm^−3^) [39,40], both displayed alongside coccoliths in figure 2. The mass of all coccoliths analysed is shown in supplementary material, figure S4. This calculation assumes a constant, calcite-equivalent electron density across growth stages; we cannot exclude a systematic change in occluded organic content with developmental stage, which the present data do not allow us to test directly. Considering, however, that the total organic content in coccoliths is approximately 2 wt% [22], we estimate that any resulting deviation from the electron density of calcite will be within the experimental error.

While coccolith volume and mass generally increased as the structure became more developed, substantial variability in values across the same growth stage causes some coccoliths at later stages of development to have lower masses than those at earlier stages, particularly evident across stages 2–3 and 3–4. In some cases, this can be explained by malformations in the coccolith, particularly at stage 4, but when no visible gaps in the structure are present, these discrepancies probably represent biological variability in the size of the scales [41]. The calculated volume and mass of the mature coccolith displayed in figure 2 were consistent with literature values calculated from X-ray nanotomography [41] and profiles estimated from SEM images [42]. We additionally observed mature intracellular coccoliths with a range of masses (supplementary material, figure S4), once again highlighting the biological variability in the size and mass during coccolith production.

By reporting changes in mass at different growth stages, we are able to provide rough estimates for the proportion of mineral in each element of the coccolith. The mineral mass of the smallest structure observed was 0.087 pg, although the rest of the coccoliths across growth stage 1 had masses ranging between 0.119 and 0.436 pg. This suggests that during the initial radial expansion of the tube element, the mineral mass of the structure increases two- to three-fold. As soon as vertical growth begins at stage 2a, the mass added to the coccolith increases substantially, reaching over 1 pg, highlighting that the highly mineralized tube element contributes nearly half of the estimated coccolith mass. The mass increase observed during stage 3a, reaching up to 1.469 pg, most likely comes from the central area elements. The last stages 3b–4 involve the full formation of shield elements, with coccoliths reaching between 2 and 3.6 pg in mass. This highlights the considerable proportion of mineral mass contained within relatively thin distal and proximal shield elements, around a third of the total coccolith weight.

Using cryo-PXCT, we also mapped the three-dimensional position of each coccolith within the cell. This revealed that coccolith location appears to be strongly dependent on its stage of growth (figure 3a–e). At the beginning of their development (stage 1), coccoliths were mostly located in a central region of the cell (red, figure 3a,b). As the coccolith developed across stage 2, scales were generally closer to the cell periphery (red, figure 3c), and by stages 3 and 4, coccoliths were always located with their convex side no further than 50 nm away from the plasma membrane (red, figure 3d–f). The nucleus of the cell, to which the coccolith vesicle is juxtaposed [38,43], did not produce enough contrast to be distinguished in the acquired electron density tomograms. Additionally, mature coccoliths were always located in regions of the cell that were not covered by external coccoliths (yellow points, figure 3d,e), even when only small gaps between two non-interlocking scales were available when the coccosphere was nearly complete (yellow points, figure 3f). This suggests that the location where exocytosis takes place is carefully controlled by the cell. Although we did not view a coccolith being exocytosed, scales are removed with their convex side facing the plasma membrane, contrasting other coccolithophore species, which expel scales in the opposite direction before they undergo extracellular rotation in order to interlock with other outward-facing scales [8].

**Figure 3.**
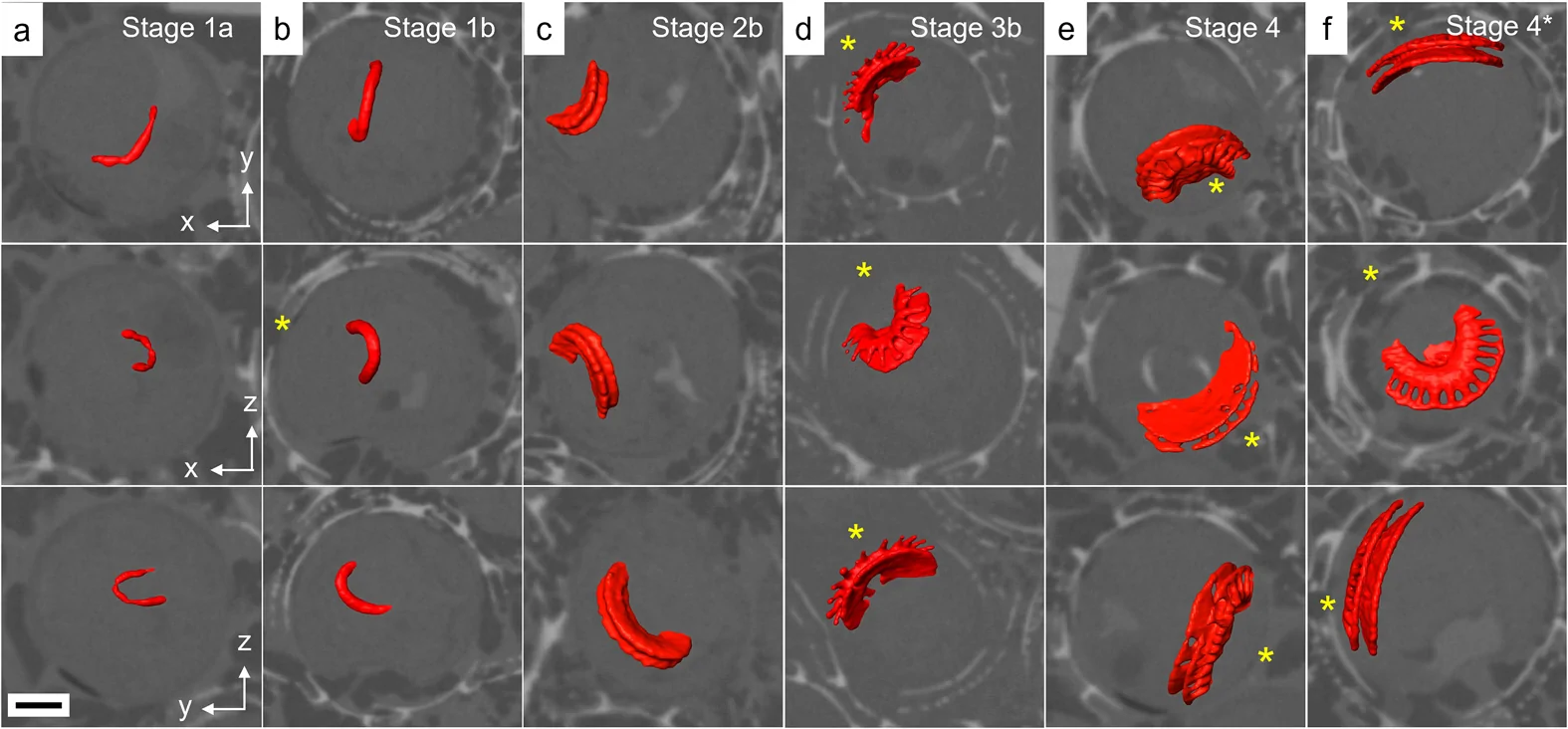
Cryo-PXCT tomograms showing cellular position of intracellular *G. huxleyi* coccoliths. (a–e) Cross-sections through the volume of six separate *G. huxleyi* cells containing coccoliths at different stages of development. Volume renderings of forming coccoliths are shown in red. In stage 1 (a,b), coccoliths are found in the centre of the cell. Between stages 2 and 4 (c–f), coccoliths are found in proximity to the membrane. Yellow markers indicate gaps in the coccosphere. Scale bar: 0.5 µm.

### 3.2. Changes in crystal morphology during the proto-coccolith ring the stage

As approximately 44% of intracellular coccoliths identified by cryo-PXCT were at growth stage 1b, it would suggest that the processes involved at this point of development are the rate-limiting step of coccolith formation. This step concerns the transition from isotropic growth of the crystals to the initial formation of the tube element, observed in stage 2a. However, the precise evolution of crystal growth cannot be resolved from cryo-PXCT tomograms. Thus, to determine the crystal morphology occurring over the early development stages, coccoliths were extracted from calcifying cells and imaged by cryoTEM and SEM. Coccoliths could be identified at each stage of growth from 1 to 2a (figure 4). Stage 1a is representative of the earliest formed protococcolith ring, comprising separated rhombohedral crystal units (figure 4a–c). Smaller units (indicated by white arrows in figure 4b) observed between larger ones may be V-units, which nucleate but do not grow any further and are overgrown by adjacent R-units. Although crystal units were not directly in contact with each other, they appear to be connected by a layer of material (arrows, figure 4c) which could be the base plate edge where crystals nucleate [16,44,45]. In stage 1b, crystal units are elongated and become interlocked (figure 4d–f), while vertical growth starts and results in the formation of the inner and outer tube elements in stage 2 (figure 4g–i).

**Figure 4.**
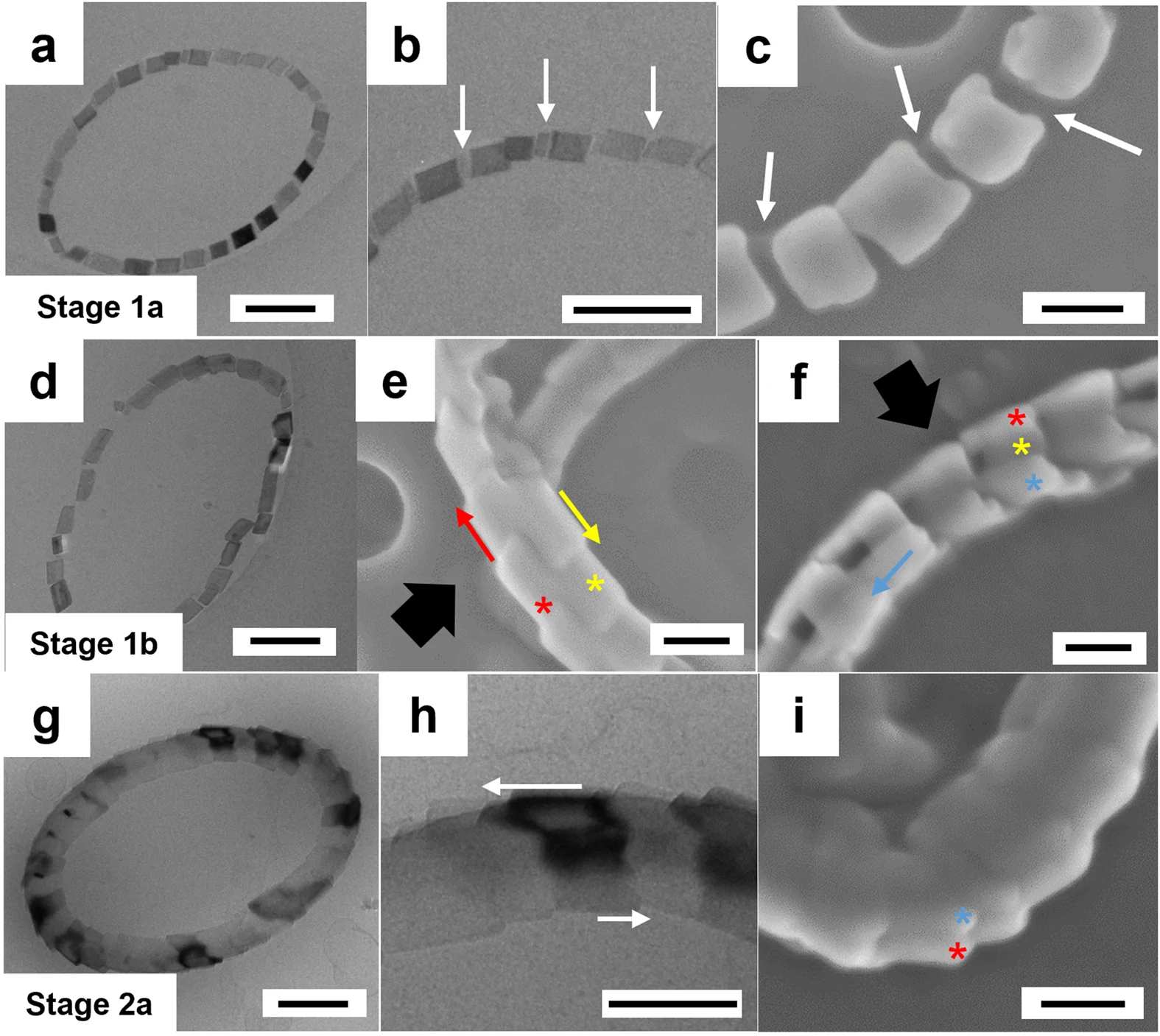
Crystal morphology and growth across stages 1 and 2a. (a) CryoTEM image of a coccolith at growth stage 1a. (b) Rhombohedral units make up the protococcolith ring, with arrows indicating smaller units present between larger ones. (c) SEM image shows that crystal units appear to be on top of an organic material, probably the base plate, indicated by arrows. (d) CryoTEM image of a coccolith at growth stage 1b. (e,f) SEM images show that crystals develop such that they interlock with adjacent units, visible when the coccolith is viewed from below (e) and above (f). Black arrows point towards the outward-facing side of the units. Outer tube and inner tube elements are highlighted by red and blue markers, respectively, with the bottom crystal face represented by yellow markers. Arrows indicate the respective direction of growth of each element. (g) CryoTEM image of a coccolith at growth stage 2a. (h) Outer and inner tube elements develop, with arrows indicating the growth direction of crystals, which causes them to overgrow the adjacent crystal. (i) The separation between the outer and inner tube, indicated, respectively, by the red and blue asterisks, can also be visualized by SEM. Scale bars: 200 nm.

The distinguishing feature of stage 1b coccoliths is that crystal morphology starts to deviate from the rhombohedral growth habit of calcite, observed in stage 1a. This change occurs because the crystals stop growing isotropically and start growing primarily in a lateral direction, overgrowing the neighbouring units. In this way, an individual unit has its horizontal bottom face outgrow the adjacent crystal in one direction (yellow arrow, figure 4e), while its outer face outgrows the unit on the other side (red arrow, figure 4e). It is noteworthy that in stage 1b, when lateral growth starts, the directions in which each crystal can grow are restricted by the presence of a neighbouring unit. It is possible, thus, that growth occurring in such a confined environment, with crystals essentially extending into each other, contributes to the reduction of the mineralization rate at this stage. Stage 1b also contains the development of the two-layered vertical faces which form the inner and outer tube elements, respectively. Two separate vertical facets are first observed during this stage (red and blue points, figure 4f), with the horizontal bottom face underneath (yellow point, figure 4f). Two distinct crystal layers are also visible once tube formation is complete at stage 2a (yellow arrows indicating separation, figure 4i). This two-layered tube element appears to function as a mechanism to keep crystals tightly interlocked around the ring [23]. It is not clear what causes the feature to develop; however, it indicates a precise control of crystal growth which could further reduce growth rate at this stage.

## 4. Discussion

Despite extensive studies of coccolith growth and its description by the widely used V/R model [14–16], key gaps remain in our understanding of the mechanisms that govern crystal growth. Resolving these requires a technique capable of imaging intracellular mineral intact, across all stages of mineralization and at sufficient throughput to capture population-level trends. PXCT meets these requirements by enabling three-dimensional imaging of intracellular CaCO₃ biominerals in their native cellular environment without disrupting the cell [14,15], while providing a large field of view that allows approximately 80 cells to be imaged simultaneously. This allowed us to image large numbers of coccoliths and track morphological evolution throughout the entire mineralization process.

In addition to providing morphological information, we could infer from the PXCT data that the mass of mature coccoliths ranges between approximately 2 and 3.6 pg. This implies that the cells must mobilize approximately 0.8–1.4 pg of calcium ions to produce one coccolith. Considering that it takes approximately 60 min for the synthesis of one coccolith, we calculate the uptake and transport of 18–30 fmol calcium ions per hour. These values are in the range of those calculated by Holtz *et al.* [46] and correspond to a flux of approximately 5 × 10^6^ calcium ions per min, as previously suggested [47]. While we have proposed that slower crystal growth kinetics are responsible for the high number of coccoliths analysed at stage 1b (discussed below), it is also worth considering that a lower calcium ion flux around this stage could be the rate-limiting factor.

The development of crystal morphology during coccolith formation largely followed the V/R model. We observed that growth roughly occurred in a single direction at one time, starting laterally after nucleation before switching to vertical to form the tube element, and finally changing to radial to produce the shield and central area elements. This suggests that growth direction can be tightly controlled by the cell, possibly through controlling the shape of the surrounding intracellular coccolith vesicle. Confining crystallization to a specific location is known to be an efficient way to control crystal morphology [48], both through physical blocking and by creating concentrated gradients which cause the preferential growth of specific crystallographic facets [25,26,49].

Cryo-PXCT revealed additional cellular control over the position of the mineralization site, enabling the location of a developing coccolith to be changed during its formation. Although it could not be resolved in the cryo-PXCT tomograms, cytoskeletal elements have been proposed to regulate the shape of the coccolith vesicle during mineralization; therefore, it is possible they could dictate the position of the vesicle as well [50,51]. Further evidence that the location of mineralization is tightly controlled was also revealed in the consistent positioning of intracellular coccoliths in regions of the cell not covered by the external coccosphere layer. The cytoskeleton has also been suggested to control exocytosis, further strengthening an argument that it could provide additional control of coccolith position to ensure that mature scales exit the cell in an empty space in the external coccosphere. In *Coccolithus braarudii*, coccoliths were shown to be secreted from a singular location, with the cell rotating within the coccosphere to ensure that the scale exited into an empty space [8]. This suggests that the mineralization site remains at a constant location, with coccosphere assembly influenced more by cell movements and the position of external coccoliths.

We were also able to observe the accumulation of coccoliths in the protococcolith ring stage, which we interpret as a rate-limiting step at the early stages of formation. At this stage, after crystals nucleate as separate rhombohedral units, growth is directed laterally to create a ring of interlocking units. This growth pattern seems to be generic across several coccolithophore species, equally observed in *C. carterae* [19,24], *C. leptoporus* [25] and *G. oceanica* [23]. Crystal growth space would be heavily constrained at this stage, both because of the proximity to the coccolith vesicle [26] and because of the presence of adjacent units that restrict the directions in which crystal morphology can develop [23,24]. Similarly, during the early stages of *G. huxleyi* formation, we observed rhombohedral units growing laterally towards each other, with the confined space created by the adjacent unit blocking certain regions and influencing where mineral can be added. This may originate from crystals nucleating at slightly offset angles to enable their organization in a ring [17,24,25], possibly causing adjacent units to block others, which prevents mineral from expanding into certain regions. Thus, one plausible explanation for the rate-limiting step observed is the slower growth kinetics when crystal growth transitions from isotropic to anisotropic while they are arranged in a constrained geometry. There are, however, other explanations which are equally likely. These include limitations in the calcium transport during this particular stage of coccolith development, as mentioned above, or the result of a biological regulatory mechanism of coccolith formation that is still unknown. Further work needs to be done to investigate the different possibilities.

A further interesting characteristic of stage 1b is the separation of the tube element into two layers, also observed in the related species *G. oceanica* directly after crystal nucleation [23]. In *G. oceanica*, the outer and inner tube elements are responsible for many structural coccolith features, growing at tilted vertical angles in opposite directions, causing adjacent crystal units to overlap and tightly interlock around the ring [23]. These features were not included in the original V/R model despite their now apparent importance to overall crystal morphology and organization. While we cannot confirm the outer and inner tubes have the same morphology in *G. huxleyi*, an assumption can be made that they form in a similar way given the comparable structures of the coccoliths produced by both species. Additionally, both the tube layers have similar morphologies in each species: the inner crystals are rhombohedral and the outer plate-like, while we also observed tightly interlocked crystals at early stages of the tube element formation in *G. huxleyi* coccoliths. Similar three-dimensional reconstruction and analysis of *G. huxleyi* coccolith structure should be conducted to confirm this configuration of crystal elements.

Coccolithophores are not just important organisms to study for their design of functional materials, but also have important applications as geological proxies for both palaeoclimate reconstructions and models for future climates. For coccolithophores to be a good proxy, a thorough understanding of coccolith mineralization is required, including knowledge of the cellular mechanisms involved in growth as well as accurate information about mineral composition and weight. Here, we provide an improved estimation of individual coccolith volume and weight at different stages of formation, which could be a valuable resource to ecological studies calculating carbon isotope ratios or determining the influence of increased CO_2_ seawater concentration on coccolithophore calcification.

## 5. Conclusions

We have provided new findings in the biomineral formation process of the coccolithophore *G. huxleyi*. The length-scale overarching combination of cryo-PXCT and electron microscopy allowed us to follow the formation of calcite coccolith scales in their native environment in three dimensions over their entire mineralization cycle. The crystal subunits assemble into a tightly packed spherical ring, where their geometric arrangement strongly influences their growth, leading to the development of crystallographic facets that reflect the presence of neighbouring subunits. Additional control appears to be imparted by controlling the position of the mineralization site in the cell, to ensure that mature coccoliths are exocytosed in the correct position to form the coccosphere. The use of spatial confinement through the use of enclosed compartments and through the control of the geometric arrangements of the crystals is one of the recurring biomineralization strategies to control mineral formation that are employed by organisms across different taxa. Therefore, our findings on how these mechanisms are expressed in coccolithophores may have implications for how biomineral formation is controlled in different tissues and species.

## Supplementary Material



## Data Availability

The X-ray computed ptychography generated in this study can be retrieved from the University of Edinburgh DataShare [52]. The remaining electron microscopy data that support the findings reported in this study are available within the paper and its supplementary material. Supplementary material is available online [53].
